# Protective effects of *Graptophyllum pictum* extract and its impact on the regulation of inflammatory mediators and short-chain fatty acids in experimental models: a multi-parameter comparative study

**DOI:** 10.3389/fvets.2026.1779196

**Published:** 2026-06-19

**Authors:** Sigit Adi Prasetyo, Endang Sri Lestari, Ignatius Riwanto, Bernardus Parish Budiono, Agung Aji Prasetyo, Bernardus Widodo Subagio, Rinaldi Hartanto, Hendry Lie, Kenny Wijaya Sutanto

**Affiliations:** 1 Division of Digestive Surgery, Department of Surgery, Faculty of Medicine, Diponegoro University/Diponegoro National Hospital, Semarang, Indonesia; 2 Department of Clinical Microbiology, Faculty of Medicine, Diponegoro University, Semarang, Indonesia; 3 Division of Pediatric Surgery, Department of Surgery, Faculty of Medicine, Diponegoro University/Diponegoro National Hospital, Semarang, Indonesia; 4 Biomedicine Science Postgraduate Program, General Surgery Resident, Faculty of Medicine, Diponegoro University, Semarang, Indonesia

**Keywords:** 5-ASA, colitis, dextran sodium sulfate, *Graptophyllum pictum*, inflammation, rat model, short-chain fatty acids

## Abstract

**Background:**

Persistent colonic inflammation disrupts epithelial integrity, alters microbial metabolites, and impairs gut function. *Graptophyllum pictum* (daun wungu) contains bioactive compounds with anti-inflammatory and mucosal-protective potential, but its impact on functional recovery and short-chain fatty acids (SCFAs) in colitis remains unclear.

**Methods:**

Thirty Wistar rats were randomized into five groups (*n* = 6/group): healthy control (K0), DSS colitis control (K–), 5-aminosalicylic acid (5-ASA, 120 mg/kg/day; K+), *G. pictum* extract (100 mg/kg twice daily; P1), and combination therapy (P2). Colitis was induced with 2% DSS for 7 days; treatments were given orally for 10 days. Primary outcomes were body weight and stool form (Bristol Stool Scale). Secondary outcomes included serum inflammatory mediators (IL-6, TNF-α, CRP, PDGF, COX-2, VEGF), fecal SCFAs (acetate, propionate, butyrate by GC-MS), leukocyte count, colonic macroscopic score, and histologic/tissue repair markers (fibroblasts, collagen density, M2 macrophages, CD34). Appropriate parametric/non-parametric tests with *post-hoc* analyses were used (α = 0.05). Ethical approval was obtained from the Faculty of Medicine, Universitas Diponegoro/Dr. Kariadi Hospital.

**Results:**

All intervention groups gained more weight than K– (*p* < 0.05), with the largest increase in P2 (Δ 16.67 ± 0.52 g). Stool consistency improved significantly in K+ and P2 (Δ −4.17 ± 0.75; post-treatment median Bristol 1–2; *p* < 0.001). Pro-inflammatory mediators differed across groups (all *p* < 0.001): compared with K–, P1/P2 markedly reduced IL-6, TNF-α, CRP, PDGF, COX-2, and VEGF. SCFAs were restored toward baseline in treated groups, with P2 showing the highest acetate/propionate/butyrate recovery vs. K– (all *p* ≤ 0.01). Macroscopic injury scores and leukocyte counts were significantly lower in K+, P1, and P2 vs. K– (*p* ≤ 0.003). Fibroblast count, collagen density, M2 macrophages, and CD34 trended upward in treatment arms but were not statistically different among groups (*p* ≥ 0.075).

**Conclusion:**

In DSS-induced colitis, *G. pictum*—especially combined with 5-ASA—improves clinical (weight, stool form) and biological outcomes by suppressing inflammatory pathways and restoring SCFA profiles, with concordant reductions in macroscopic damage and leukocytosis. These data support *G. pictum* as a promising adjunct to standard therapy for promoting gut homeostasis and functional recovery.

## Introduction

Persistent inflammation of the colonic mucosa disrupts nutrient absorption and intestinal motility, leading to progressive body weight loss, diarrhea, and metabolic imbalance (1). These physiological alterations reflect the breakdown of the guts structural and microbial equilibrium, where epithelial integrity, immune tone, and microbial metabolites are intimately linked (1). Among the most critical metabolites are short-chain fatty acids (SCFAs), acetate, propionate, and butyrate, which are produced through bacterial fermentation of dietary fibers (2). SCFAs sustain colonocyte energy metabolism, regulate mucin and tight junction synthesis, and suppress inflammatory signaling. When inflammation reduces SCFA production, mucosal permeability increases, stool consistency deteriorates, and systemic nutritional recovery becomes impaired (3–5). Hence, monitoring parameters such as body weight, stool characteristics, and SCFA profiles provides an integrated view of intestinal health and functional healing.

Body weight and stool form represent accessible yet meaningful indicators of gastrointestinal function (6). Weight restoration signals improvement in metabolic absorption and energy utilization, while stool normalization reflects balanced water reabsorption, microbiota stability, and neuromuscular regulation of colonic transit (7, 8). Experimental models such as Dextran Sodium Sulfate (DSS)-induced colitis closely replicate the epithelial erosion, cytokine activation, and microbial dysbiosis seen in clinical intestinal inflammation (9). In such conditions, assessing both metabolic and microbial recovery offers greater translational relevance than histological endpoints alone, as they reflect the physiological return to gut homeostasis.

*G. pictum* (L.) Griff., a traditional Indonesian medicinal plant known as “daun wungu,” contains bioactive flavonoids, alkaloids, and phenolic compounds with demonstrated antioxidant, anti-inflammatory, and mucosal-protective activities (10). Its potential to modulate immune responses and enhance epithelial regeneration has been reported in various inflammatory models, yet its impact on gut functional recovery and microbial metabolite balance remains unexplored (10). Understanding how *G. pictum* influences SCFA production, stool characteristics, and postoperative metabolic adaptation could offer a novel therapeutic perspective that integrates microbiota regulation with host tissue repair. Therefore, this study investigated the effects of *G. pictum* extract on body weight, stool characteristics, and SCFA profiles in DSS-induced colitis rats to elucidate its potential role in restoring gut homeostasis and promoting functional recovery.

## Methods

This experimental study was designed to evaluate the effects of *Graptophyllum pictum* extract on body weight, stool characteristics, and short-chain fatty acid (SCFA) profiles in Wistar rats with dextran sodium sulfate (DSS)-induced colitis. The experimental protocol was approved by the Ethics Committee of the Faculty of Medicine, Universitas Diponegoro/Dr. Kariadi Hospital, Semarang, Indonesia. All procedures involving animals were conducted in accordance with the guidelines established by the National Academy of Sciences, as outlined in the Guide for the Care and Use of Laboratory Animals (11).

Fresh leaves of *Graptophyllum pictum* were collected, extraction was performed using a conventional maceration technique following standard pharmacognostic procedures. The powdered plant material was soaked in 70% ethanol at ambient temperature for 72 h with occasional stirring to enhance mass transfer and extraction efficiency. The selected extraction duration falls within the commonly recommended maceration period of 3–7 days. After extraction, the mixture was filtered, and the filtrate was concentrated under reduced pressure using a rotary evaporator to yield the crude extract.

A total of thirty Wistar rats were randomly allocated into five groups: normal control (K0), negative control (K–), positive control (K+), treatment group 1 (P1), and treatment group 2 (P2). The K0 group received no colitis induction or treatment. The K– group was induced with colitis using dextran sodium sulfate (DSS) without therapeutic intervention, while the K+ group received standard therapy with 5-aminosalicylic acid (5-ASA) at a dose of 120 mg/kg/day. The P1 group was administered *G. pictum* extract at 100 mg/kg twice daily, and the P2 group received a combination of *G. pictum* extract (100 mg/kg twice daily) and 5-ASA (120 mg/kg/day). All treatments were delivered orally via gavage for 10 days following colitis induction.

The sample size (*n* = 6 per group) was determined based on prior studies employing DSS-induced colitis models, in which comparable group sizes were sufficient to detect significant differences in inflammatory and metabolic outcomes. An a priori power analysis using G^*^Power 3.1, assuming an effect size (f) of 0.6, a significance level (α) of 0.05, and a statistical power (1–β) of 0.80, indicated a minimum requirement of five animals per group. To account for potential attrition during DSS induction, six animals per group were included.

Colitis was induced by administering a 2% DSS solution *ad libitum* for seven consecutive days. This model was selected for its ability to replicate key pathological features of human ulcerative colitis, including epithelial disruption, mucosal ulceration, and inflammatory activation, which manifest clinically as diarrhea, hematochezia, and weight loss. Throughout the study, animals were maintained under controlled environmental conditions (temperature 22–25 °C, humidity 50%−60%, 12-h light–dark cycle) with free access to standard chow and water. Body weight and stool consistency were recorded before and after the intervention period.

Stool consistency was evaluated using an adapted Bristol Stool Scale (BSS) as a semi-quantitative measure of fecal characteristics in the DSS-induced rat model. Although the Bristol Stool Scale was originally developed for human clinical assessment, it has been utilized in experimental gastrointestinal studies as a practical indicator of stool consistency and intestinal functional status. Adaptation for the animal model was based on stool appearance, shape, and water content characteristics observed during sample collection. Lower scores represented firmer and more normalized stool consistency, whereas higher scores represented loose or diarrheal stool patterns. Stool assessments were independently performed by two investigators blinded to group allocation to minimize observer bias.

At the conclusion of the experiment (day 25), fresh fecal samples were collected and immediately stored at −80 °C until analysis. For short-chain fatty acid (SCFA) analysis, approximately 100 mg of fecal sample was homogenized in distilled water and acidified with hydrochloric acid to facilitate extraction. Samples were vortexed and centrifuged at 10,000 rpm for 10 min at 4 °C, and the supernatant was collected for analysis. Acetate, propionate, and butyrate concentrations were quantified using gas chromatography–mass spectrometry (GC–MS). Separation was performed using a capillary column under helium carrier gas with controlled temperature programming. Samples were analyzed under electron ionization conditions, and compound identification was performed by comparison with reference standards and spectral libraries. Quantification was achieved using calibration curves generated from known standard concentrations. Final SCFA concentrations were expressed as mean ± standard deviation (SD).

All analyses were performed under standardized laboratory conditions to ensure consistency and reproducibility of measurements.

## Statistical analysis

Data distribution was assessed using the Shapiro–Wilk test. Normally distributed data were analyzed using one-way ANOVA followed by LSD *post-hoc* tests. Non-normally distributed data were analyzed using the Kruskal–Wallis test followed by Dunn's *post-hoc* test with Bonferroni correction. Data are presented as mean ± SD for parametric variables and median (interquartile range) for non-parametric variables. A *p-value* < 0.05 was considered statistically significant.

## Histopathological analysis

Colonic tissue samples were fixed in 10% formalin, embedded in paraffin, and stained with hematoxylin–eosin (H&E). Microscopic evaluation included epithelial integrity, inflammatory infiltration, and mucosal architecture. Representative images from each group are provided, and semi-quantitative scoring was performed.

## Results

All experimental groups exhibited an increase in body weight following the intervention, with statistically significant differences observed between groups (*p* < 0.05). The normal control group (K0) demonstrated the greatest mean weight gain (Δ = 18.50 ± 0.84 g; 95% CI: 17.62–19.38), whereas the DSS-induced untreated group (K–) showed the lowest increase (Δ = 8.17 ± 0.98 g; 95% CI: 7.14–9.20). Treatment groups displayed intermediate improvements, with K+ (Δ = 12.83 ± 0.41 g; 95% CI: 12.40–13.26), P1 (Δ = 12.33 ± 0.52 g; 95% CI: 11.78–12.88), and P2 (Δ = 16.67 ± 0.52 g; 95% CI: 16.12–17.22), indicating enhanced metabolic recovery, particularly in the combination therapy group. The quantitative findings are summarized in Tables 1–3, while representative macroscopic and histopathological findings are presented in Figures 1–23.

**Table 1:** Comparison of body weight among study groups.

Group	Pre (Mean ±SD)	Post (Mean ±SD)	Median (min–max)	Δ (Mean ±SD)	Median Δ (min–max)	*p* (pre–post)	*Post-hoc* (Post)	*Post-hoc* (Δ)
K0	190.50 ± 3.27	209.00 ± 4.05	210.5 (203–213)	18.50 ± 0.84	19 (17–19)	0.024^*^	K– < 0.001^*^	0.003^*^
							K+ 0.004^*^	
							P1 0.003^*^	
							P2 0.052	
K–	188.67 ± 2.73	196.83 ± 2.64	197 (193–200)	8.17 ± 0.98	8.5 (7–9)	0.026^*^	K+ 0.005^*^	0.003^*^
							P1 0.006^*^	
							P2 < 0.001 ^*^	
K+	190.00 ± 3.41	202.83 ± 3.37	203 (197–207)	12.83 ± 0.41	13 (12–13)	0.020^*^	P1 0.933	0.002^*^
							P2 0.279	
P1	190.33 ± 2.16	202.67 ± 2.16	202.5 (200–206)	12.33 ± 0.52	12 (12–13)	0.023^*^	P2 0.244	0.003^*^
P2	188.33 ± 4.27	205.00 ± 4.24	205 (200–210)	16.67 ± 0.52	17 (16–17)	0.023^*^	—	0.003^*^

^*^Statistically significant difference (*p* < 0.05).

**Table 2:** Comparison of fecal characteristics (Bristol Stool Scale) among study groups.

Group	Pre (Mean ±SD)	Post (Mean ±SD)	Median (min–max)	Δ (Mean ±SD)	Median Δ (min–max)	*p* (pre–post)	*Post-hoc* (Post)	*Post-hoc* (Δ)
K0	1.00 ± 0.00	1.00 ± 0.00	1 (1–1)	0.00 ± 0.00	0.00 (0–0)	1.000	K– 0.002^*^	0.018^*^
							K+ 0.002^*^	
							P1 0.002^*^	
							P2 0.002^*^	
K–	5.67 ± 0.52	4.50 ± 0.55	4.5 (4–5)	−1.17 ± 0.75	−1 (−2 to 0)	0.013^*^	K+ 0.002^*^	< 0.001^*^
							P1 0.011^*^	
							P2 0.002 ^*^	
K+	5.33 ± 0.52	1.17 ± 0.41	1 (1–2)	−4.17 ± 0.75	−4 (−5 to −3)	< 0.001^*^	P1 0.003^*^	< 0.001^*^
							P2 1.000	
P1	5.50 ± 0.55	3.17 ± 0.75	3 (2–4)	−2.33 ± 1.21	−2.5 (−4 to −1)	0.005^*^	P2 0.003^*^	0.001^*^
P2	5.33 ± 0.52	1.17 ± 0.41	1 (1–2)	−4.17 ± 0.75	−4 (−5 to −3)	< 0.001^*^	—	< 0.001^*^

^*^Statistically significant difference (*p* < 0.05).

**Table 3:** Comparative analysis of inflammatory mediators, SCFAs, macroscopic scores, and tissue repair parameters across experimental groups (mean ± SD).

Group	Mean ±SD	Median (min–max)	*p-value*	*Post-hoc* comparisons
IL-6 (pg/mL)				
K0	19.33 ± 3.94	19.34 (14.33–24.33)	< 0.001^*^	K– < 0.001^*^ K+ < 0.001^*^ P1 < 0.001^*^ P2 < 0.001^*^
K–	167.39 ± 10.51	168.50 (154.33–179.33)		K+ < 0.001^*^ P1 < 0.001^*^ P2 < 0.001^*^
K+	86.28 ± 5.31	85.17 (81–96)		P1 0.002^*^ P2 < 0.001^*^
P1	103.50 ± 5.45	102.67 (97.67–112.67)		P2 < 0.001^*^
P2	49.33 ± 5.77	48.50 (42.67–57.67)		
TNF-α (pg/mL)				
K0	4.90 ± 0.23	4.88 (4.55–5.19)	< 0.001^*^	K– < 0.001^*^ K+ < 0.001^*^ P1 < 0.001^*^ P2 < 0.001^*^
K–	21.82 ± 0.69	21.87 (21.05–22.62)		K+ < 0.001^*^ P1 < 0.001^*^ P2 < 0.001^*^
K+	10.70 ± 0.40	10.78 (10.17–11.17)		P1 < 0.001^*^ P2 < 0.001^*^
P1	12.42 ± 0.38	12.38 (11.88–12.94)		P2 < 0.001^*^
P2	7.69 ± 0.44	7.65 (7.18–8.39)		
VEGF (pg/mL)				
K0	9.83 ± 0.50	9.83 (9.20–10.46)	< 0.001^*^	K– < 0.001^*^ K+ < 0.001^*^ P1 < 0.001^*^ P2 < 0.001^*^
K–	49.30 ± 1.32	49.44 (47.65–50.80)		K+ < 0.001^*^ P1 < 0.001^*^ P2 < 0.001^*^
K+	17.22 ± 0.85	17.18 (16.13–18.45)		P1 < 0.001^*^ P2 < 0.001^*^
P1	25.63 ± 1.02	25.70 (24.33–26.85)		P2 < 0.001^*^
P2	12.77 ± 0.66	12.67 (11.93–13.82)		
CRP (mg/dL)				
K0	0.76 ± 0.02	0.76 (0.74–0.79)	< 0.001^*^	K– < 0.001^*^ K+ < 0.001^*^ P1 < 0.001^*^ P2 < 0.001^*^
K–	2.57 ± 0.03	2.57 (2.52–2.60)		K+ < 0.001^*^ P1 < 0.001^*^ P2 < 0.001^*^
K+	1.59 ± 0.03	1.59 (1.56–1.63)		P1 < 0.001^*^ P2 < 0.001^*^
P1	1.80 ± 0.02	1.80 (1.77–1.83)		P2 < 0.001^*^
IL-6 (pg/mL)				
P2	0.99 ± 0.01	0.99 (0.98–1.00)		
PDGF (pg/mL)				
K0	1.32 ± 0.03	1.32 (1.29–1.37)	< 0.001^*^	K– < 0.001^*^ K+ < 0.001^*^ P1 < 0.001^*^ P2 0.006^*^
K–	8.24 ± 0.28	8.24 (7.77–8.63)		K+ < 0.001^*^ P1 < 0.001^*^ P2 < 0.001^*^
K+	1.95 ± 0.09	1.99 (1.81–2.03)		P1 0.054 P2 < 0.001^*^
P1	2.11 ± 0.05	2.11 (2.04–2.17)		P2 < 0.001^*^
P2	1.56 ± 0.04	1.55 (1.52–1.62)		
COX-2 (pg/mL)				
K0	45.44 ± 8.28	44.34 (36.00–59.33)	< 0.001^*^	K– < 0.001^*^ K+ < 0.001^*^ P1 < 0.001^*^ P2 < 0.001^*^
K–	424.89 ± 7.20	424.34 (416.00–436.00)		K+ < 0.001^*^ P1 < 0.001^*^ P2 < 0.001^*^
K+	142.11 ± 8.80	142.67 (129.33–152.67)		P1 < 0.001^*^ P2 < 0.001^*^
P1	184.33 ± 8.10	184.34 (172.67 – 196.00)		P2 < 0.001^*^
P2	85.45 ± 8.80	86.00 (72.67 – 96.00)		—
Acetate (μmol/g feces)				
K0	90.40 ± 31.62	81.71 (58.08–150.88)	< 0.001^*^	K– < 0.001^*^ K+ < 0.001^*^ P1 < 0.001^*^ P2 < 0.001^*^
K–	20.66 ± 2.75	19.86 (17.93–25.40)		K+ 0.007^*^ P1 0.566 P2 0.002^*^
K+	45.74 ± 3.42	46.24 (41.32–50.05)		P1 0.026^*^ P2 < 0.001^*^
P1	25.61 ± 2.21	25.63 (22.50–29.07)		P2 0.007^*^
P2	50.79 ± 8.08	50.25 (42.39–65.63)		
Propionate (μmol/g feces)				
K0	34.53 ± 11.01	31.78 (22.74–55.46)	< 0.001^*^	K– < 0.001^*^ K+ < 0.001^*^ P1 < 0.001^*^ P2 < 0.001^*^
K–	8.49 ± 1.23	8.04 (7.22–10.59)		K+ 0.005^*^ P1 0.699 P2 0.001^*^
K+	17.71 ± 1.20	17.59 (15.92–19.18)		P1 < 0.001^*^ P2 0.464
P1	9.66 ± 1.05	9.73 (8.31–11.11)		P2 0.002^*^
P2	19.94 ± 3.09	19.67 (17.06–25.60)		
Butyrate (μmol/g feces)				
K0	12.07 ± 3.92	11.42 (8.02–19.43)	< 0.001^*^	K– 0.004^*^ K+ 0.004^*^ P1 0.004^*^ P2 0.006^*^
K–	2.94 ± 0.80	3.14 (1.65–3.76)		K+ 0.004^*^ P1 0.873 P2 0.004^*^
K+	5.80 ± 0.52	5.75 (5.16–6.57)		P1 0.055 P2 0.078
P1	3.45 ± 1.91	2.59 (2.33–7.27)		P2 0.025^*^
P2	6.98 ± 1.35	6.90 (5.45–9.25)		
Macroscopic score				
K0	0.17 ± 0.41	0 (0–1)	< 0.001^*^	K– 0.003^*^ K+ 0.005^*^ P1 0.002^*^ P2 0.180
K–	5.83 ± 0.75	6 (5–7)		K+ 0.003^*^ P1 0.003^*^ P2 0.003^*^
K+	1.67 ± 0.82	1.5 (1–3)		P1 0.084 P2 0.155
P1	2.67 ± 1.03	2 (2–4)		P2 0.016^*^
P2	0.83 ± 0.98	0.5 (0–2)		—
Leukocyte (cells/μL)				
K0	79.50 ± 13.40	75 (67–103)	< 0.001 ^*^	K– < 0.001^*^ K+ 0.001^*^ P1 < 0.001^*^ P2 0.038^*^
K–	248.50 ± 34.66	250 (201–298)		K+ 0.014^*^ P1 0.039^*^ P2 < 0.001^*^
K+	180.33 ± 57.68	171 (98–275)		P1 0.657 P2 0.102
P1	192.00 ± 57.86	197.5 (103–281)		P2 0.042^*^
P2	136.33 ± 44.99	125 (82–191)		—
Fibroblast (cells/high-power field)				
K0	17.00 ± 8.58	15.5 (8–29)	0.086	—
K–	14.33 ± 9.52	12 (6–32)		—
K+	18.33 ± 10.52	18 (7–35)		—
P1	25.17 ± 2.79	24.5 (22–29)		—
P2	27.33 ± 10.86	25.5 (13–45)		—
Collagen (%)				
K0	18.79 ± 10.20	21.81 (4.46–29.35)	0.740	—
K–	17.88 ± 3.39	17.38 (13.82–23.12)		—
K+	19.21 ± 11.41	15.08 (6.86–38.92)		—
P1	17.73 ± 6.21	17.91 (9.30–26.17)		—
P2	23.99 ± 10.83	24.67 (12.18–40.91)		—
M2 (positive cells/high-power field)				
K0	17.83 ± 7.31	15 (12–32)	0.075	—
K–	16.00 ± 9.70	12 (7–29)		—
K+	21.33 ± 14.05	17.5 (7–40)		—
P1	35.67 ± 18.28	32 (19–69)		—
P2	27.33 ± 15.54	23.5 (14–58)		—
CD34 (positive cells/high-power field)				
K0	21.67 ± 8.07	23.5 (8–32)	0.199	—
K–	11.67 ± 6.15	10 (5–20)		—
K+	13.50 ± 5.17	14.5 (7–20)		—
P1	15.17 ± 8.75	12 (8–31)		—
P2	12.33 ± 8.91	9.5 (6–30)		—

^*^Statistically significant difference (*p* < 0.05).

**Figure 1:**
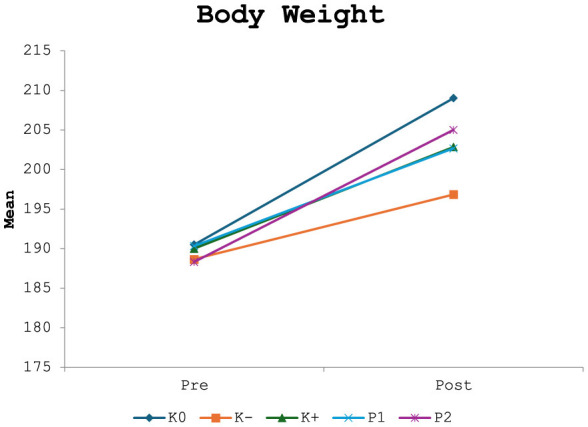
Line graph showing differences regarding body weight among study groups.

**Figure 2:**
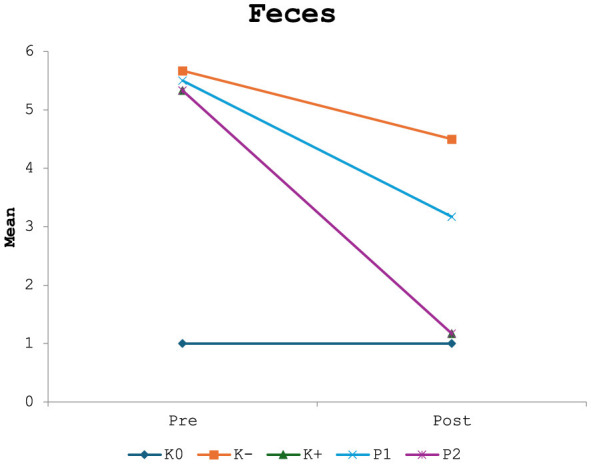
Line graph showing Bristol stool scale among study groups.

**Figure 3:**
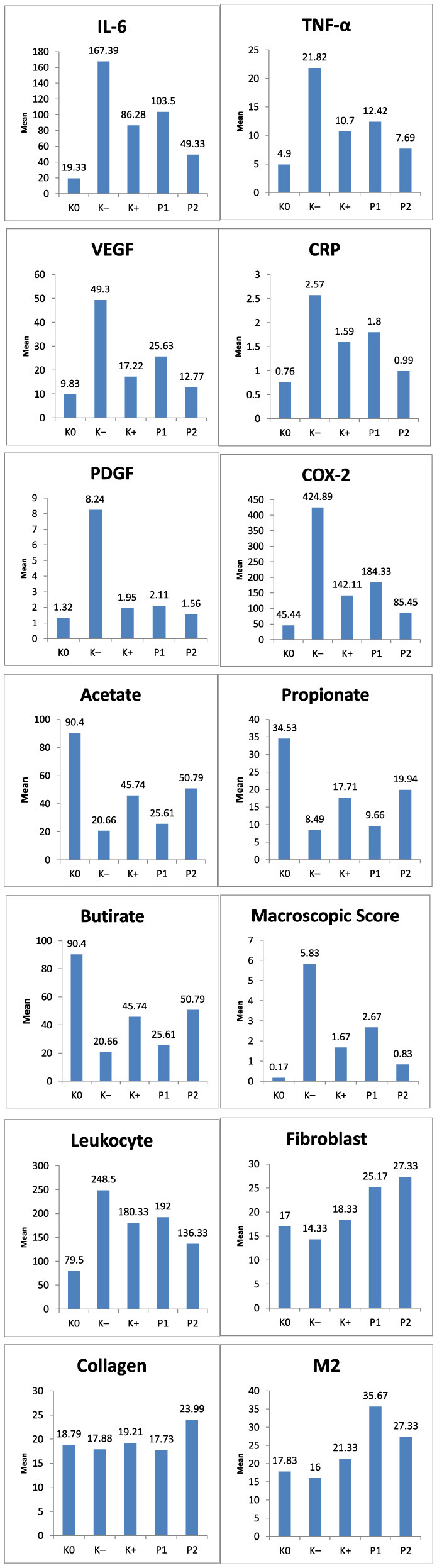
Bar graph showing inflammatory the effect of SCFA across experimental groups.

**Figure 4:**
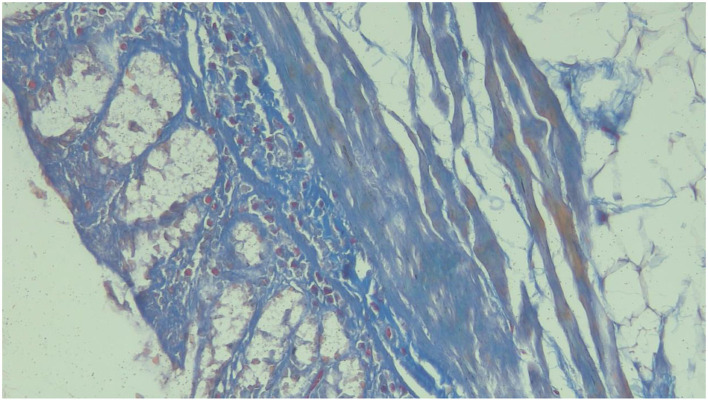
Representative photomicrograph of colonic tissue from the K0 (normal control) group stained with Mallory–Aniline blue. The section demonstrates preserved mucosal architecture with intact crypt structures, well-organized collagen fibers (blue), and minimal inflammatory cell infiltration. Original magnification: 400 × .

**Figure 5:**
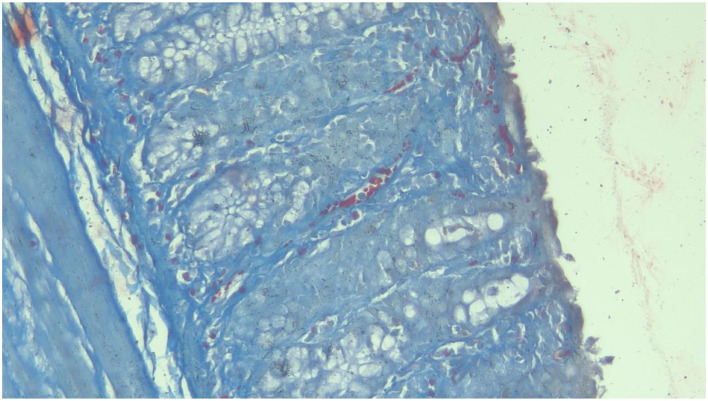
Representative photomicrograph of colonic tissue from the K– (negative control, DSS-induced colitis) group stained with Mallory–Aniline blue. The section shows disrupted mucosal architecture with distorted crypt structures, increased inflammatory cell infiltration, and disorganized collagen fibers (blue). Areas of epithelial damage and inflammatory changes are evident. Original magnification: 400 × .

**Figure 6:**
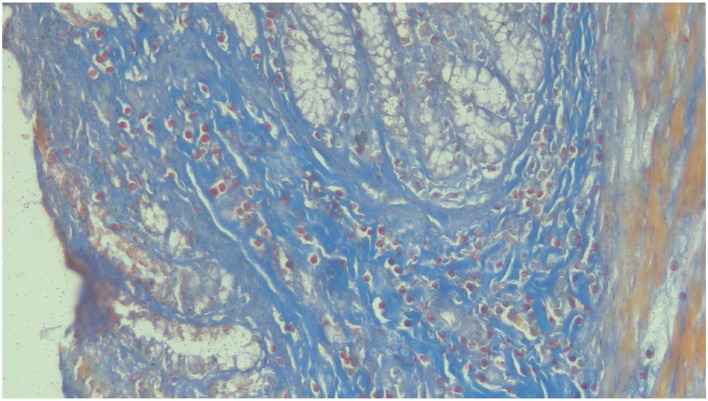
Representative photomicrograph of colonic tissue from the K+ (positive control, 5-ASA-treated) group stained with Mallory–Aniline blue. The section demonstrates partially restored mucosal architecture with improved crypt organization, reduced inflammatory cell infiltration, and more structured collagen fibers (blue) compared to the untreated colitis group. Original magnification: 400 × .

**Figure 7:**
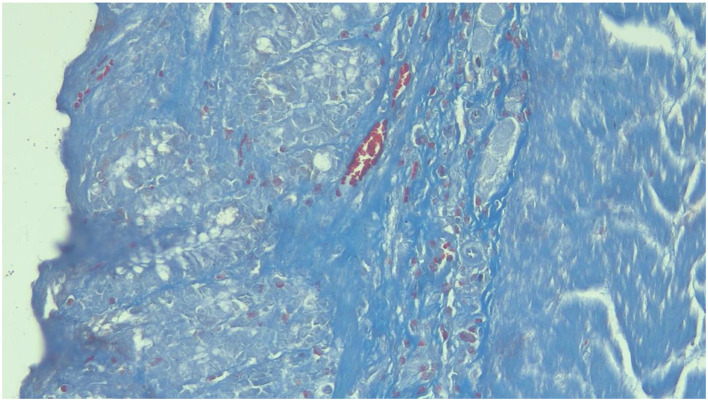
Representative photomicrograph of colonic tissue from the P1 group (*Graptophyllum pictum* extract-treated) stained with Mallory–Aniline blue. The section shows improved mucosal architecture with relatively preserved crypt structures, reduced inflammatory cell infiltration, and more organized collagen fibers (blue), indicating partial tissue recovery. Original magnification: 400 × .

**Figure 8:**
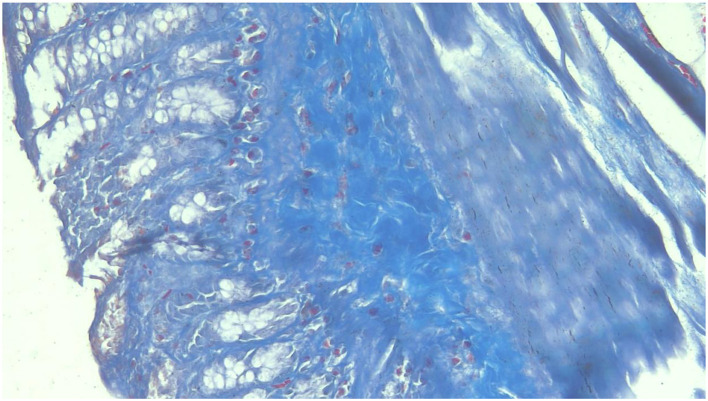
Representative photomicrograph of colonic tissue from the P2 group (*Graptophyllum pictum* extract + 5-ASA-treated) stained with Mallory–Aniline blue. The section demonstrates near-normal mucosal architecture with well-preserved crypt structures, minimal inflammatory cell infiltration, and densely organized collagen fibers (blue), indicating advanced tissue recovery. Original magnification: 400 × .

**Figure 9:**
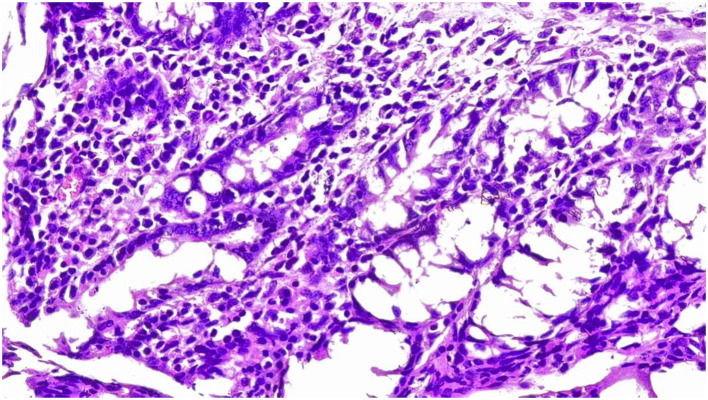
Representative photomicrograph of colonic tissue stained with hematoxylin and eosin (H&E). The section shows disrupted mucosal architecture with distorted crypts, marked inflammatory cell infiltration, and areas of epithelial damage, consistent with active colitis. Original magnification: 400 × .

**Figure 10:**
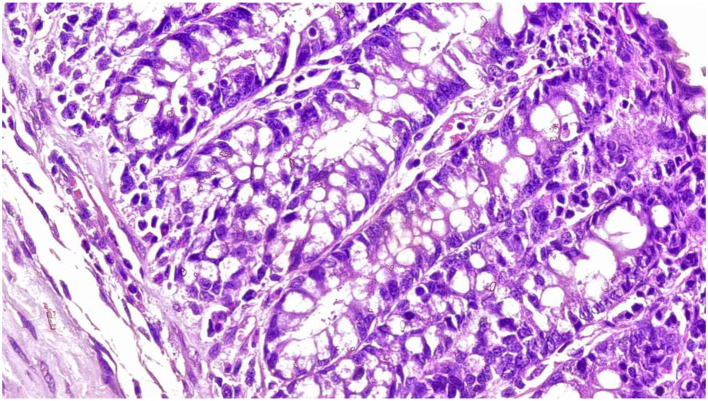
Representative photomicrograph of colonic tissue stained with hematoxylin and eosin (H&E). The section demonstrates preserved crypt structures with abundant goblet cells, mild inflammatory cell infiltration in the lamina propria, and relatively intact mucosal architecture, suggesting partial restoration of colonic mucosa. Original magnification: 400 × .

**Figure 11:**
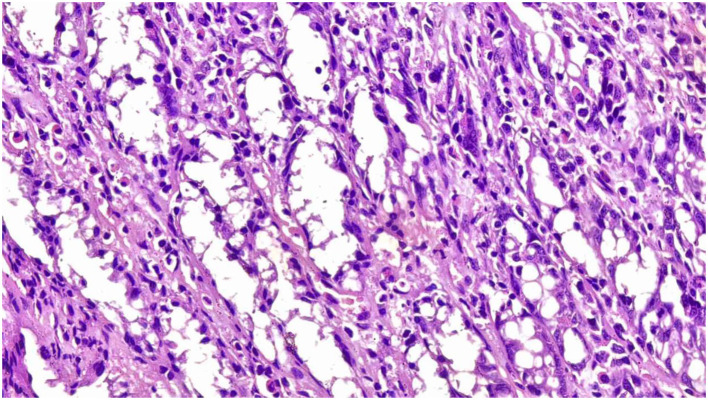
Representative photomicrograph of colonic tissue from the K+ group stained with hematoxylin and eosin (H&E). The section shows disrupted mucosal architecture with irregular and elongated crypts, goblet cell depletion, moderate inflammatory cell infiltration within the lamina propria, and focal epithelial injury, consistent with active colonic inflammation. Original magnification: 400 × .

**Figure 12:**
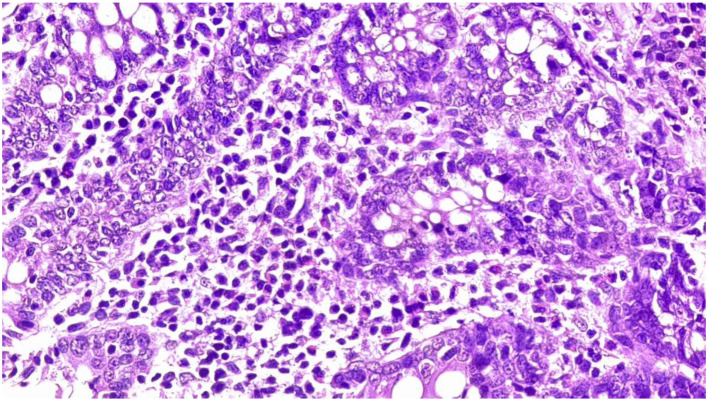
Representative photomicrograph of colonic tissue from the P1 group stained with hematoxylin and eosin (H&E). The section demonstrates moderate disruption of mucosal architecture with irregular crypt arrangement, focal goblet cell depletion, and prominent inflammatory cell infiltration within the lamina propria. Areas of epithelial degeneration are also observed, indicating partial but incomplete improvement of colonic inflammation. Original magnification: 400 × .

**Figure 13:**
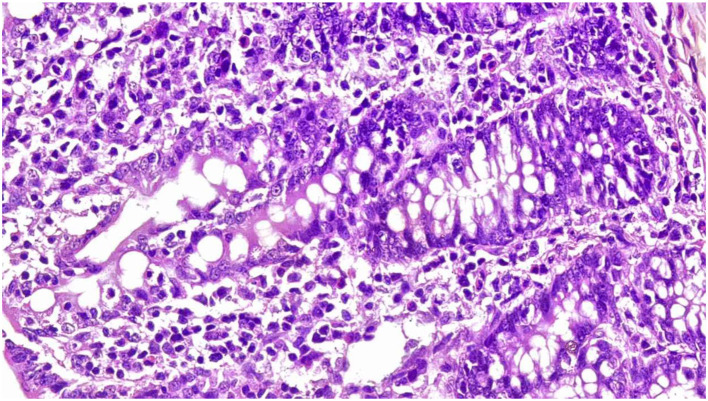
Representative photomicrograph of colonic tissue from the P2 group stained with hematoxylin and eosin (H&E). The section demonstrates partial restoration of mucosal architecture with more organized crypt structures and preserved goblet cells. However, moderate inflammatory cell infiltration and focal epithelial disruption are still present within the lamina propria, indicating ongoing but reduced colonic inflammation. Original magnification: 400 × .

**Figure 14:**
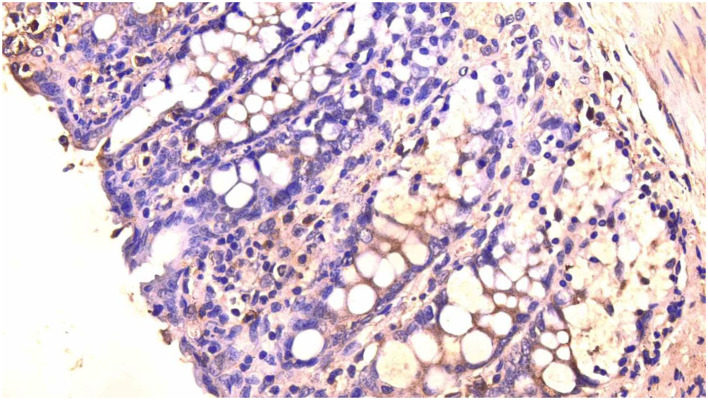
Representative immunohistochemical photomicrograph of colonic tissue from the K0 group stained for CD163. The section demonstrates positive brown cytoplasmic immunoreactivity in scattered stromal inflammatory cells consistent with CD163-expressing macrophages, with preserved colonic crypt architecture and minimal inflammatory infiltration. Original magnification: 400 × .

**Figure 15:**
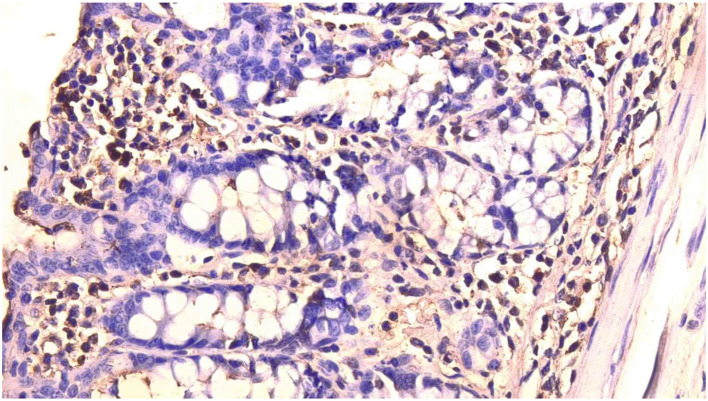
Representative immunohistochemical photomicrograph of colonic tissue from the K– group stained for CD163. The section demonstrates increased brown cytoplasmic immunoreactivity in numerous inflammatory stromal cells consistent with elevated CD163-expressing macrophage infiltration, accompanied by disrupted mucosal architecture and inflammatory cell accumulation. Original magnification: 400 × .

**Figure 16:**
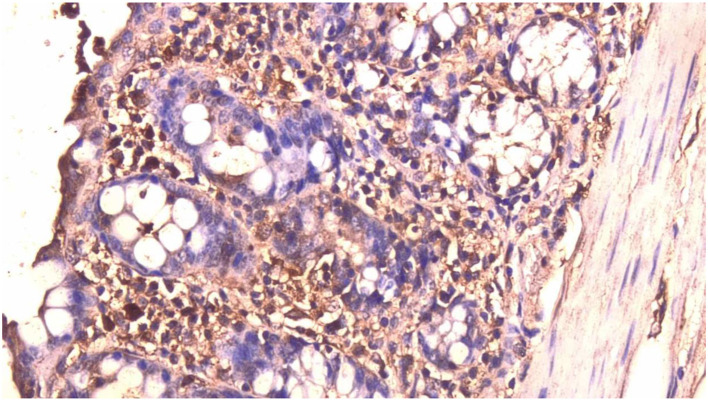
Representative immunohistochemical photomicrograph of colonic tissue from the K^+^ group stained for CD163. The section shows prominent brown cytoplasmic immunoreactivity in numerous stromal inflammatory cells, consistent with increased CD163-expressing macrophages. Colonic crypt architecture is largely preserved, with mild to moderate inflammatory cell infiltration. Original magnification: 400 × .

**Figure 17:**
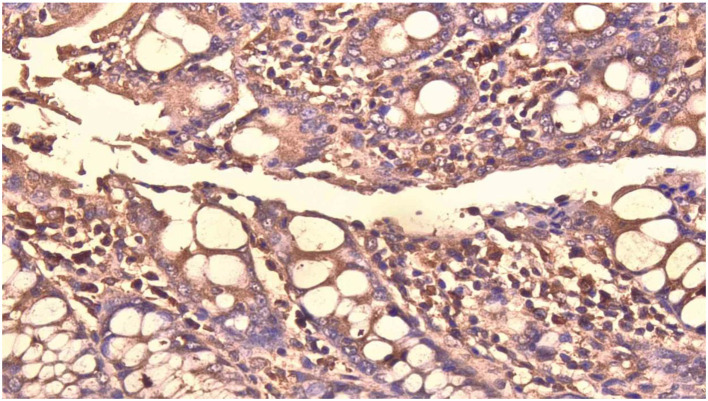
Representative immunohistochemical photomicrograph of colonic tissue from the P1 group stained for CD163. The section demonstrates moderate brown cytoplasmic immunoreactivity in stromal inflammatory cells, indicating the presence of CD163-positive macrophages. Colonic crypt architecture is preserved, with mild inflammatory cell infiltration in the lamina propria. Original magnification: 400 × .

**Figure 18:**
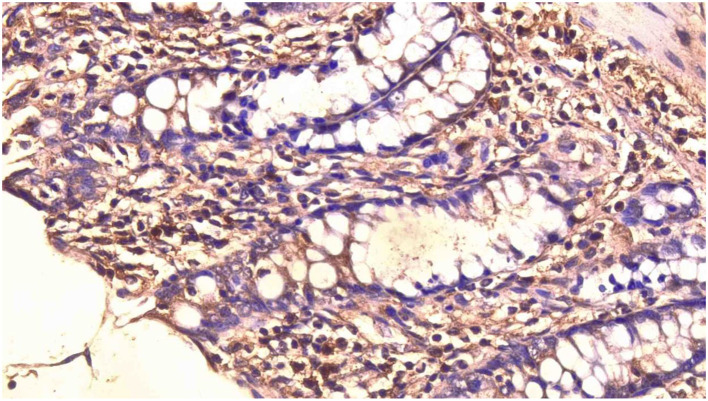
Representative immunohistochemical photomicrograph of colonic tissue from the P2 group stained for CD163. The section demonstrates increased brown cytoplasmic immunoreactivity in stromal inflammatory cells, indicating a higher density of CD163-positive macrophages compared to P1. There is more prominent inflammatory cell infiltration within the lamina propria, with mild-to-moderate distortion of crypt architecture and areas of goblet cell depletion. Original magnification: 400 × .

**Figure 19:**
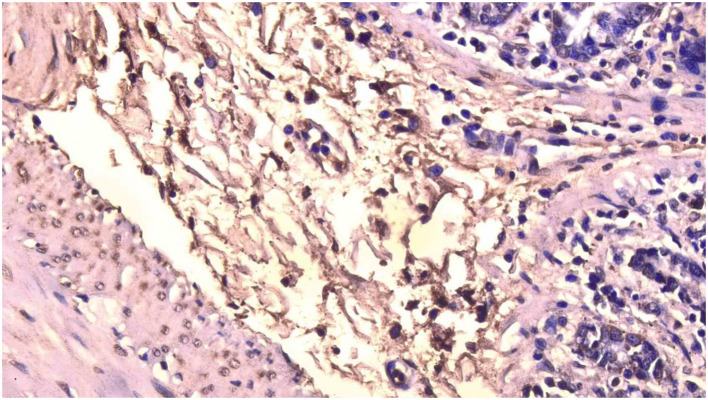
Representative immunohistochemical photomicrograph of colonic tissue from the K0 group stained for CD34. The section shows minimal to weak brown membranous and cytoplasmic immunoreactivity in endothelial cells, indicating low baseline microvascular density. Colonic crypt architecture is well preserved, with no significant stromal vascular proliferation. Original magnification: 400 × .

**Figure 20:**
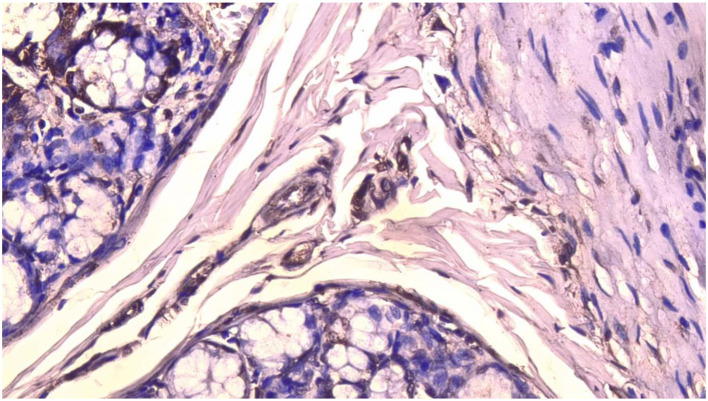
Representative immunohistochemical photomicrograph of colonic tissue from the K– group stained for CD34. The section demonstrates sparse brown immunoreactivity in endothelial cells, reflecting limited angiogenic activity. The mucosal architecture remains intact, with mild stromal cellularity and minimal vascular expansion. Original magnification: 400 × .

**Figure 21:**
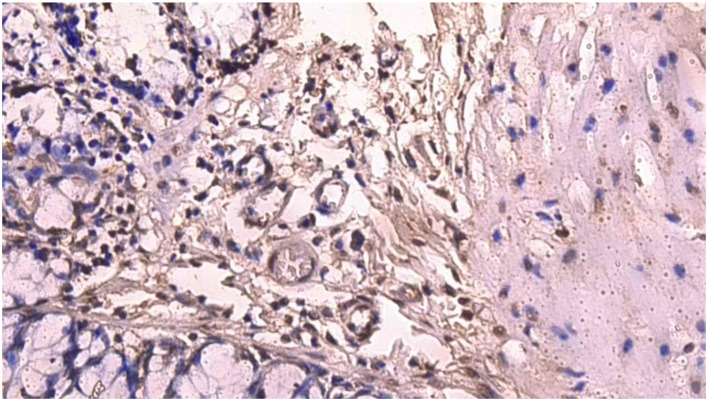
Representative immunohistochemical photomicrograph of colonic tissue from the K+ group stained for CD34. The section shows increased brown endothelial staining, indicating enhanced microvascular density and active angiogenesis within the stroma. This is accompanied by mild to moderate inflammatory infiltration and early vascular proliferation. Original magnification: 400 × .

**Figure 22:**
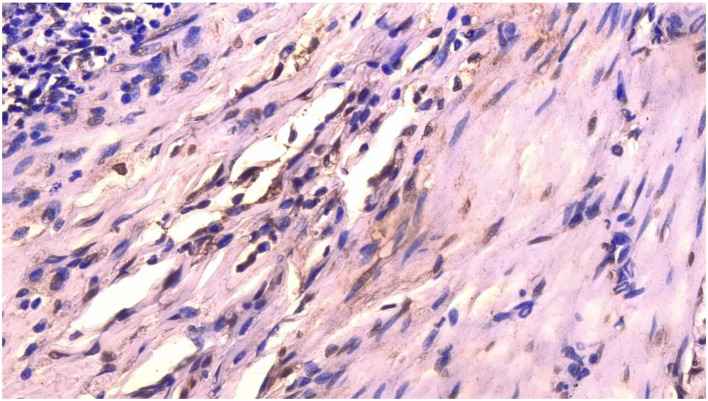
Representative immunohistochemical photomicrograph of colonic tissue from the P1 group stained for CD34. The section demonstrates prominent and diffuse brown immunoreactivity in endothelial cells, consistent with marked angiogenesis and increased microvascular density. Stromal expansion and inflammatory cell infiltration are evident, while overall crypt architecture remains relatively preserved. Original magnification: 400 × .

**Figure 23:**
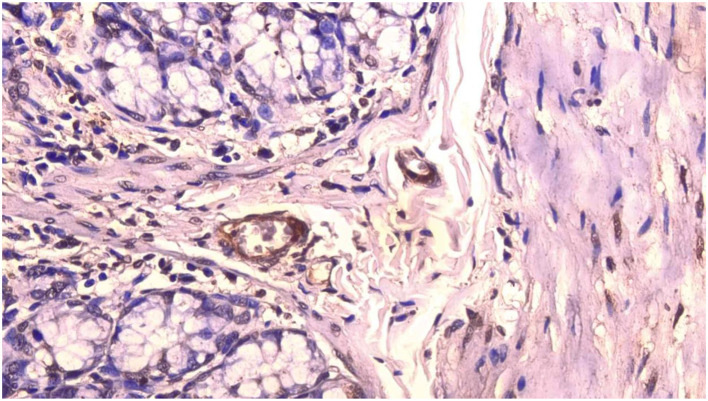
Representative immunohistochemical photomicrograph of colonic tissue from the P2 group stained for CD34. The section shows increased brown endothelial staining, indicating enhanced microvascular density and active angiogenesis within the stroma. This is accompanied by mild to moderate inflammatory infiltration and early vascular proliferation. Original magnification: 400 × .

Stool consistency improved significantly across all intervention groups, as assessed by the Bristol Stool Scale (*p* < 0.001). The most pronounced improvement was observed in the K+ and P2 groups (Δ = −4.17 ± 0.75; 95% CI: −4.96 to −3.38), with post-treatment scores approaching normal values. The P1 group showed moderate improvement (Δ = −2.33 ± 1.21; 95% CI: −3.60 to −1.06), while the K– group exhibited only minimal change (Δ = −1.17 ± 0.75; 95% CI: −1.96 to −0.38), indicating persistent dysfunction in untreated colitis.

Serum inflammatory mediators differed significantly among all groups (*p* < 0.001). IL-6 levels were markedly elevated in the K– group (167.39 ± 10.51 pg/mL; 95% CI: 156.35–178.43) compared with the K0 group (19.33 ± 3.94 pg/mL; 95% CI: 15.19–23.47). Treatment resulted in substantial reductions, particularly in P2 (49.33 ± 5.77 pg/mL; 95% CI: 43.27–55.39), followed by K+ (86.28 ± 5.31 pg/mL; 95% CI: 80.71–91.85) and P1 (103.50 ± 5.45 pg/mL; 95% CI: 97.78–109.22). A similar pattern was observed for TNF-α, where K– showed the highest levels (21.82 ± 0.69; 95% CI: 21.10–22.54), while treatment groups demonstrated significant reductions, most notably in P2 (7.69 ± 0.44; 95% CI: 7.23–8.15).

VEGF concentrations were also significantly elevated in the K– group (49.30 ± 1.32; 95% CI: 47.91–50.69) compared to K0 (9.83 ± 0.50; 95% CI: 9.30–10.36), indicating increased angiogenic activity under inflammatory conditions. Treatment groups showed marked reductions, with P2 demonstrating near-normalization (12.77 ± 0.66; 95% CI: 12.08–13.46). Similarly, CRP levels were significantly higher in K– (2.57 ± 0.03; 95% CI: 2.54–2.60) compared to K0 (0.76 ± 0.02; 95% CI: 0.74–0.78), while P2 exhibited substantial reduction (0.99 ± 0.01; 95% CI: 0.98–1.00), indicating attenuation of systemic inflammation.

PDGF and COX-2 levels followed comparable trends, with the highest expression observed in the K– group (PDGF: 8.24 ± 0.28; 95% CI: 7.95–8.53; COX-2: 424.89 ± 7.20; 95% CI: 417.33–432.45). Treatment groups demonstrated significant reductions, particularly in P2 (PDGF: 1.56 ± 0.04; 95% CI: 1.52–1.60; COX-2: 85.45 ± 8.80; 95% CI: 76.21–94.69), indicating effective suppression of inflammatory and repair-associated signaling pathways.

Fecal short-chain fatty acid (SCFA) concentrations differed significantly across groups (*p* < 0.001), reflecting microbial metabolic alterations. Acetate levels were highest in K0 (90.40 ± 31.62; 95% CI: 57.21–123.59) and markedly reduced in K– (20.66 ± 2.75; 95% CI: 17.77–23.55). Treatment groups showed restoration toward baseline, particularly P2 (50.79 ± 8.08; 95% CI: 42.30–59.28). A similar pattern was observed for propionate and butyrate, where K– exhibited the lowest concentrations (propionate: 8.49 ± 1.23; 95% CI: 7.20–9.78; butyrate: 2.94 ± 0.80; 95% CI: 2.10–3.78), while P2 showed the greatest recovery (propionate: 19.94 ± 3.09; 95% CI: 16.69–23.19; butyrate: 6.98 ± 1.35; 95% CI: 5.56–8.40).

Macroscopic injury scores were significantly higher in K– (5.83 ± 0.75; 95% CI: 5.04–6.62) compared to K0 (0.17 ± 0.41; 95% CI: −0.26–0.60), confirming severe colonic damage. Treatment groups demonstrated progressive improvement, with the lowest scores observed in P2 (0.83 ± 0.98; 95% CI: −0.20–1.86), consistent with gross morphological recovery. Leukocyte counts followed a similar trend, with K– showing marked elevation (248.50 ± 34.66; 95% CI: 212.11–284.89), while treatment groups exhibited reduced counts, particularly P2 (136.33 ± 44.99; 95% CI: 89.09–183.57), indicating attenuation of systemic inflammatory response.

Histological parameters, including fibroblast count, collagen density, M2 macrophage expression, and CD34 levels, did not differ significantly among groups (*p* > 0.05), although treatment groups showed a consistent upward trend. For instance, fibroblast counts increased from 17.00 ± 8.58 (95% CI: 7.99–26.01) in K0 to 27.33 ± 10.86 (95% CI: 15.92–38.74) in P2, while M2 macrophages rose from 17.83 ± 7.31 (95% CI: 10.15–25.51) to 27.33 ± 15.54 (95% CI: 11.02–43.64). These findings suggest ongoing tissue repair and remodeling, although statistical significance was not reached, likely due to variability and limited sample size.

## Discussion

All treatment groups in this study demonstrated significant increases in body weight, with the P2 group exhibiting the greatest gain (Δ = 16.67 ± 0.52 g, *p* = 0.023), compared to the negative control group (Δ = 8.17 ± 0.98 g, *p* = 0.026). These findings indicate enhanced metabolic recovery and improved nutritional status following combined therapy in the P2 group. Consistent with these results, Kim et al. (11) reported a 20%−25% restoration in body weight in DSS-induced colitic mice (*p* < 0.001). Similarly, Firoozi et al. (12) observed significant weight recovery in patients with ulcerative colitis treated with sodium butyrate (*p* < 0.05), supporting the role of microbiota-derived metabolites in improving systemic metabolic outcomes (11, 12). In contrast, weight recovery stagnated with dysbiosis, as noted by Chassaing et al. (13), indicating the dependency on microbiota. The observed restoration of SCFA concentrations in treatment groups may be associated with improved intestinal recovery. Previous studies have suggested that SCFAs can contribute to colonocyte metabolism and maintenance of epithelial barrier integrity; however, these mechanisms were not directly evaluated in the present study. In addition, previous studies have proposed that SCFAs may influence metabolic regulation through activation of G protein–coupled receptors such as GPR41 and GPR43. However, receptor signaling pathways were not examined in the present study and should therefore be interpreted as potential mechanisms rather than experimentally demonstrated findings. Collectively, these mechanisms may underlie the improved nutritional status and metabolic recovery observed in the P2 group (14, 15). Collectively, the present findings, together with existing literature, indicate a strong association between SCFA availability in the colonic mucosa and the restoration of metabolic function, as well as the promotion of mucosal repair (15). The observed increase in CD34 expression and restoration of SCFA concentrations may reflect enhanced tissue repair, neovascularization, and metabolic recovery, consistent with previous reports (42, 43).

The observed significant weight gain in the treatment groups is consistent with evidence indicating that SCFAs facilitate the restoration of intestinal nutrient absorption by attenuating inflammation and oxidative stress. Supporting this, Saygili et al. (16) demonstrated that high-fiber dietary interventions in inflamed colonic models resulted in significant weight gain (*p* = 0.002). These findings are in line with the present results, where the improvements observed in the P1 and P2 groups positively correlate with increased body weight, further underscoring the role of SCFAs in promoting metabolic recovery (16). Conversely, Borrego-Ruiz and Borrego (17) reported that SCFA-deficient microbial models exhibited inconsistent weight outcomes, highlighting the critical role of host–microbiome interactions in regulating energy utilization and metabolic homeostasis. Specifically, butyrate and acetate function as key signaling molecules that regulate energy homeostasis by promoting mitochondrial biogenesis and modulating catabolic cytokine activity, partly through interactions with insulin-like growth factor-1 (IGF-1) pathways. The significant weight gain observed in the P1–P2 groups suggests restoration of microbial metabolite production alongside adaptive resolution of inflammation. Repletion of SCFAs is closely associated with improved energy utilization and enhanced epithelial regeneration, ultimately contributing to recovery from inflammation-induced weight loss.

In the present study, stool consistency improved markedly, particularly in the K+ and P2 groups (Δ = −4.17 ± 0.75; *p* < 0.001). This finding indicates normalization of stool form and improved colonic transit function. These results are consistent with those reported by Tarrerias et al. (18), who demonstrated a reduction in stool scores from 6 to 3 within 5 days (*p* = 0.01), as well as Zhao et al. (19), who showed that SCFA-mediated regulation enhances epithelial sodium channel activity, reduces intestinal fluid loss, and contributes to normalization of stool consistency (*p* < 0.05) (18, 19). In contrast, Priyadarshini et al. (20) reported that disruption of SCFA availability and impaired microbial fermentation were associated with prolonged diarrhea in inflammatory bowel disease models. These findings further underscore the critical role of SCFAs in maintaining microbial metabolic function and intestinal fluid balance. These improvements may be attributed to the physiological effects of SCFAs, which enhance epithelial barrier integrity by promoting tight junction assembly and suppressing pro-secretory cytokines such as TNF-α and IL-8. Restoration of stool consistency and reduction of intestinal fluid loss are likely mediated by butyrate's histone deacetylase (HDAC)–inhibitory activity, which upregulates mucin production and enhances epithelial sodium absorption, thereby contributing to improved colonic fluid balance and barrier function (21). Therefore, consistent with previous studies, SCFAs play a central role in restoring intestinal fluid balance and epithelial transport function.

The restoration of intestinal motility and barrier function is reflected in the fecal normalization observed across the treatment groups (22). This finding is consistent with existing literature demonstrating that activation of GPR43 by acetate and propionate regulates colonic fluid absorption and motility, while previous evidence suggests that butyrate may modulate inflammatory pathways involving HDAC inhibition and NF-κB regulation. Since these signaling pathways were not directly assessed in this study, such mechanisms remain hypothetical explanations for the observed findings (23). Supporting evidence from Tazoe et al. (24) further indicates that SCFA receptor activation in enteroendocrine L-cells stimulates the release of peptide YY (PYY), which reduces intestinal secretion and modulates motility. Although some experimental models report variability in responsiveness depending on SCFA concentration or microbial composition, the present findings align with the prevailing evidence that functional stool recovery is closely associated with the restoration of colonic SCFA levels (25). Mechanistically, this process involves enhanced epithelial regeneration, stabilization of tight junctions, suppression of secretory signaling, and re-establishment of mucosal homeostasis, collectively contributing to improved fecal consistency.

## Interleukin-6 (IL-6)

The present study demonstrated a significant elevation in IL-6 levels across the experimental groups, indicating the presence of an active inflammatory response. The negative control (K–) group exhibited the highest IL-6 concentration, reflecting pronounced cytokine upregulation under pathological conditions. The marked difference between the K– and K0 groups confirms the sensitivity of IL-6 as a biomarker of tissue injury and immune activation. In contrast, the lower IL-6 levels observed in the P1 and P2 groups suggest a modulatory effect associated with therapeutic intervention.

Mechanistically, IL-6 exerts its effects primarily through the JAK/STAT signaling pathway and plays a central role in inducing C-reactive protein (CRP) synthesis in hepatocytes. The significant reductions observed in this study (*post-hoc p* < 0.001 between most groups) indicate effective attenuation of the IL-6–CRP axis. These findings are consistent with previous reports by Shahini and Shahini (26) and Li et al. (27), which demonstrated that modulation of IL-6 signaling is associated with improved inflammatory control and tissue recovery. The significant difference observed between the P1 and P2 groups suggests that either prolonged duration or combination therapy may enhance cytokine modulation. These findings further support the role of IL-6 as both a diagnostic biomarker and a therapeutic target in inflammation-related pathophysiology.

## Tumor necrosis factor-alpha (TNF-α)

Significant variations in TNF-α levels among the groups confirm robust modulation of the inflammatory response. The elevated concentration observed in the negative control (K–) group indicates acute pro-inflammatory activation, likely contributing to tissue injury. In contrast, the reduced TNF-α levels in the P1 and P2 groups suggest that the observed reductions in inflammatory markers are consistent with attenuation of inflammatory activity following treatment. The statistical significance observed across nearly all intergroup comparisons underscores TNF-α as a key biomarker of inflammation.

TNF-α is a central mediator of immune cell recruitment and cytokine amplification (28). The lower levels detected in the post-treatment groups reflect effective suppression of inflammatory signaling pathways. These findings further suggest that the interventions may inhibit TNF-α production at the transcriptional and/or translational level, thereby contributing to improved inflammatory control (28).

## Vascular endothelial growth factor (VEGF)

VEGF levels varied significantly across experimental conditions, indicating dynamic regulation of angiogenic processes. The negative control (K–) group exhibited the highest VEGF expression, likely reflecting tissue stress and compensatory angiogenesis in response to injury. In contrast, the reduced VEGF levels observed in the K+, P1, and P2 groups suggest that therapeutic interventions contributed to stabilization of vascular responses and limitation of pathological neovascularization. The significant differences among all groups underscore the sensitivity of VEGF to both injury and repair stimuli (29). VEGF plays a critical role in regulating vascular permeability, endothelial cell proliferation, and wound healing. Elevated VEGF expression in the disease model is consistent with hypoxia-driven signaling pathways, whereas the moderate levels observed in treatment groups indicate partial restoration of vascular homeostasis. This balanced modulation may support effective tissue repair while preventing excessive angiogenic activity associated with chronic inflammation (29).

## C-reactive protein (CRP)

CRP levels demonstrated distinct variations among the experimental groups, with a clear pattern of elevation in the untreated condition. The significantly increased CRP concentration in the negative control (K–) group reflects systemic inflammation consistent with activation of the acute-phase response. In contrast, the lower CRP levels observed in the P1 and P2 groups indicate a reduced systemic inflammatory burden following treatment. The consistent statistical significance (*p* < 0.001) across intergroup comparisons supports the reliability of CRP as a sensitive biomarker of therapeutic efficacy (30). CRP is synthesized by hepatocytes in response to pro-inflammatory cytokine signaling, particularly interleukin-6 (IL-6). The parallel reduction in both IL-6 and CRP levels in the treatment groups reinforces the mechanistic link between cytokine suppression and attenuation of systemic inflammation. These findings suggest that the applied interventions may effectively modulate hepatic acute-phase protein synthesis, contributing to improved inflammatory control (30).

## Platelet-derived growth factor (PDGF)

PDGF levels were markedly elevated in the negative control (K–) group compared to both control and treatment groups, suggesting an active tissue repair response following inflammation. Increased PDGF expression in inflammatory conditions is associated with fibroblast recruitment and extracellular matrix deposition. The observed reduction in PDGF levels in the P1 and P2 groups may indicate progression toward tissue recovery and resolution of the acute reparative phase. The significant differences among groups further highlight the role of PDGF in the transition from inflammation to tissue repair (31).

PDGF is a critical regulator of fibroblast proliferation, angiogenesis, and wound remodeling. The lower concentrations observed in the treated groups suggest attenuation of fibroproliferative signaling, consistent with a shift toward tissue stabilization. This decline may reflect advancement to a later stage of healing, in which active repair processes have subsided. Therefore, the PDGF expression patterns observed in this study illustrate the temporal dynamics of tissue repair and their modulation by therapeutic intervention (31).

## Cyclooxygenase-2 (COX-2)

COX-2 expression was significantly upregulated in the negative control (K–) group, indicating robust inflammatory activation. This enzyme is closely associated with prostaglandin synthesis and the amplification of local inflammatory responses. The observed reduction in COX-2 levels in the K+, P1, and P2 groups suggests effective suppression of pro-inflammatory pathways following therapeutic intervention. These differences are consistent with the expected downregulation of inflammatory mediators in response to treatment (32).

As an inducible enzyme, COX-2 plays a central role in mediating pain and inflammation through the production of prostanoids derived from the arachidonic acid pathway. Its marked reduction in the treatment groups indicates inhibition of this pathway, potentially leading to decreased prostaglandin synthesis and attenuation of edema and leukocyte infiltration. Therefore, modulation of COX-2 expression represents a key mechanism underlying the anti-inflammatory effects observed in this experimental model (32).

## Acetate concentration

Acetate levels differed significantly across all groups, indicating dynamic metabolic modulation in response to both disease and therapeutic intervention. The normal control (K0) group exhibited the highest baseline concentration, whereas the negative control (K–) group showed a marked reduction, reflecting disrupted microbial metabolism under inflammatory conditions. Intermediate acetate levels observed in the K+, P1, and P2 groups suggest partial restoration of short-chain fatty acid (SCFA) balance following treatment (33).

Acetate, as a predominant SCFA, plays a central role in energy homeostasis and immune regulation. The decreased levels observed during inflammation may result from impaired microbial fermentation or increased metabolic consumption. In contrast, the elevated concentrations in the treatment groups indicate recovery of microbial activity and normalization of fermentation processes. These findings support the association between gut microbiota restoration and the attenuation of systemic inflammatory responses (33).

## Propionate concentration

Propionate concentrations varied significantly among the groups, demonstrating a pattern similar to that observed for acetate. The negative control (K–) group exhibited the lowest mean levels, whereas the post-treatment groups showed a gradual restoration of propionate concentrations. These findings suggest recovery of microbial fermentation capacity and short-chain fatty acid (SCFA) production following intervention. The observed pattern further supports an association between SCFA normalization and the resolution of inflammation (34).

Propionate plays a key role in regulating lipid metabolism and immune signaling through activation of G protein–coupled receptors. Its restoration in the treated groups indicates improved microbial activity and enhanced intestinal barrier integrity. Increased propionate levels may contribute to suppression of pro-inflammatory cytokines and maintenance of epithelial homeostasis. Overall, these results reflect the beneficial metabolic effects of the intervention on gut-associated immune balance (34).

## Butyrate concentration

Butyrate levels were significantly altered across experimental conditions, with the lowest concentrations observed in the inflamed negative control (K–) group. This reduction likely reflects impairment of butyrate-producing microbiota and/or increased metabolic utilization during inflammatory states. The restoration of butyrate levels in the P1 and P2 groups indicates recovery of microbial fermentation capacity following therapeutic intervention. The significant differences observed among all groups further highlight the sensitivity of butyrate to both inflammatory disruption and treatment effects (35).

Butyrate plays a central role in colonocyte energy metabolism and the regulation of intestinal inflammation. The increased levels observed in the treatment groups suggest concurrent recovery of both microbial activity and epithelial function. As a histone deacetylase (HDAC) inhibitor, butyrate influences gene expression by suppressing pro-inflammatory cytokine production. Therefore, the pattern observed in this study supports the potential of butyrate as a functional biomarker of intestinal health restoration and mucosal healing (35).

## Macroscopic score

Macroscopic examination revealed distinct differences among the experimental groups, consistent with the biochemical findings. The negative control (K–) group exhibited the highest macroscopic injury scores, whereas the normal control (K0) group showed minimal alterations. Progressive reductions in injury scores observed in the K+, P1, and P2 groups provide visual evidence of tissue recovery following intervention. These findings highlight the concordance between gross pathological changes and underlying molecular parameters (36).

Macroscopic assessment offers a direct evaluation of visible inflammation and tissue damage. The observed alignment between macroscopic improvement and reductions in inflammatory biomarkers further supports the consistency and reliability of the treatment effects. Notably, the lower scores in the P2 group suggest near-complete resolution of inflammatory lesions. Overall, these results indicate that morphological healing parallels biochemical normalization, reinforcing the therapeutic potential of the interventions (36).

## Leukocyte count

Leukocyte levels were markedly elevated in the negative control (K–) group, indicating active immune cell infiltration and an ongoing inflammatory response. In contrast, the substantial reductions observed in the P1 and P2 groups demonstrate effective attenuation of inflammation following treatment. The K+ and K0 groups exhibited intermediate leukocyte levels, suggesting a more regulated immune response. These patterns support the role of leukocyte count as a reliable indicator of systemic inflammatory status (37).

Leukocyte infiltration reflects both acute immune activation and tissue repair processes. The observed decrease following treatment may be attributed to suppression of chemotactic signaling and reduced cytokine-mediated recruitment of immune cells. This reduction likely contributes to decreased tissue damage and facilitates improved healing outcomes. Overall, these findings indicate that modulation of leukocyte dynamics plays a critical role in the resolution of inflammation (37).

## Fibroblast count

Fibroblast counts did not differ significantly among the experimental groups, although slightly higher mean values were observed in the treatment groups. These findings suggest a relatively stable fibroblast presence during the tissue recovery phase. The comparable counts in the K– and K0 groups indicate that fibroblast activity was not markedly altered by inflammatory conditions. In contrast, the modest increases observed in the P1 and P2 groups may reflect gradual extracellular matrix deposition during the healing process (38).

Fibroblasts play a critical role in collagen synthesis and tissue remodeling. The stability of fibroblast numbers across groups may indicate a balanced and regulated repair response. Although inflammation typically promotes fibroblast recruitment, the absence of excessive proliferation suggests controlled tissue regeneration. These observations are consistent with the stabilization of PDGF expression and collagen-related parameters, supporting an overall coordinated healing process (38).

## Collagen density

Collagen levels did not differ significantly among the experimental groups, indicating a relatively uniform pattern of extracellular matrix deposition across conditions. Although the P2 group exhibited the highest mean value, statistical analysis confirmed the absence of significant differences. This consistency suggests that collagen remodeling occurred at a comparable level regardless of treatment (39).

Collagen synthesis is a key component of tissue repair and structural restoration. The lack of significant variation implies that all groups achieved a similar degree of collagen deposition during the recovery phase. It is possible that the interventions influenced the quality or organization of collagen rather than its overall quantity. Therefore, the structural integrity of tissue repair appears to have been maintained consistently across all experimental groups (39).

## M2 macrophages

M2 macrophage levels did not demonstrate significant differences among the experimental groups, although the treatment groups exhibited numerically higher mean values. The relatively lower levels observed in the K0 and K– groups suggest limited macrophage polarization during the active inflammatory phase, whereas the increased M2 counts in the P1 and P2 groups may reflect enhanced activity during the resolution phase. This trend is consistent with the progression toward anti-inflammatory signaling and tissue recovery. M2 macrophages are closely associated with tissue regeneration and immune regulation, and their elevated presence in the treated groups suggests a shift from pro-inflammatory (M1) to pro-repair (M2) phenotypes. Such phenotypic switching is critical for restoring tissue homeostasis following acute inflammation. Although the differences were not statistically significant, the observed trend supports the role of the interventions in promoting effective immune modulation (40).

## CD34 expression

CD34 expression remained statistically comparable across all experimental groups, indicating stable angiogenic and progenitor cell activity throughout the study period. The slightly higher expression observed in the K0 group is consistent with baseline vascular integrity, while only minimal variations were noted among the other groups. This overall consistency suggests that progenitor-mediated repair mechanisms were not substantially altered by inflammatory or treatment conditions. CD34 serves as a marker for endothelial progenitor and stem cells involved in neovascularization, and its relatively unchanged expression implies a preserved regenerative capacity across groups. These findings indicate that vascular repair processes remained stable despite the presence of inflammation (41).

This study utilized a single dose of *G. pictum* extract based on prior literature; however, dose–response relationships were not evaluated. This limits the ability to determine the optimal therapeutic dose or assess potential toxicity thresholds. Future studies should incorporate multiple dosing regimens to better characterize pharmacodynamic effects.

## Study limitation

This study has several limitations. First, the relatively small sample size may reduce statistical power and limit the generalizability of the findings. Second, only a single dose of *Graptophyllum pictum* extract was evaluated, precluding assessment of dose–response relationships. Third, comprehensive phytochemical profiling was not performed, limiting the identification of specific active compounds responsible for the observed effects. Fourth, the 10-day treatment duration primarily reflects acute-phase responses and may not adequately capture long-term mucosal remodeling. Fifth, histopathological evaluation did not include specialized staining techniques, such as Alcian blue, which could provide additional insight into mucin production, goblet cell preservation, and mucosal barrier integrity. Finally, SCFA measurements were restricted to fecal concentrations and did not assess systemic absorption or distribution. Future studies should address these limitations to provide a more comprehensive understanding of the therapeutic potential and mechanisms of action of *G. pictum*.

Furthermore, although substantial changes in inflammatory mediators and SCFA concentrations were observed, molecular signaling pathways potentially involved in these responses, including GPR signaling, NF-κB activation, and HDAC-related mechanisms, were not directly assessed. Therefore, mechanistic interpretations should be considered hypothesis-generating and based primarily on prior literature rather than direct experimental evidence from the present study.

## Conclusion

This study provides a comprehensive evaluation of the effects of *Graptophyllum pictum* extract on inflammatory pathways, metabolic function, and tissue repair in a DSS-induced colitis model. The findings demonstrate that inflammation induces substantial alterations in cytokine profiles, growth factors, and short-chain fatty acid (SCFA) metabolism, while therapeutic intervention—particularly combination therapy—effectively attenuates these disruptions. The consistent reduction in pro-inflammatory mediators alongside the restoration of SCFA levels highlights a coordinated interaction between immune modulation and microbial metabolic recovery.

Importantly, this study integrates multiple dimensions of assessment, including clinical indicators (body weight and stool characteristics), biochemical markers, microbial metabolites, macroscopic evaluation, and histological parameters, providing a multidimensional perspective on intestinal inflammation and recovery. Although certain regenerative markers did not reach statistical significance, their consistent upward trends suggest the initiation of tissue repair processes that may become more evident with longer observation periods or larger sample sizes.

While limitations remain, including sample size, duration of intervention, and the absence of detailed phytochemical and dose–response analyses, this work represents a substantial step toward understanding the therapeutic potential of *Graptophyllum pictum*. We anticipate that these findings will serve as a foundational reference for future experimental and translational studies.

Furthermore, this study underscores the potential of indigenous medicinal plants as accessible and biologically active therapeutic agents. In the Indonesian context, where biodiversity is exceptionally rich, such research is essential to support the scientific validation and development of natural products for the management of complex and severe diseases. We hope that this work will stimulate further large-scale investigations and contribute to the advancement of evidence-based phytotherapy in Indonesia.

## Data Availability

The original contributions presented in the study are included in the article/supplementary material, further inquiries can be directed to the corresponding author/s.
